# Canalicular Adenomas of Minor Salivary Glands: A Systematic Review of Case Reports and Case Series

**DOI:** 10.3390/jcm15124655

**Published:** 2026-06-16

**Authors:** Mohamed Jaber, Nadin Abouseif, Mawada Abdelmagied, Alaa Mohamed El-Ameen

**Affiliations:** 1 Department of Clinical Sciences, College of Dentistry, Ajman University, Ajman P.O. Box 346, United Arab Emirates; m.abdelmagied@ajman.ac.ae; 2 Center of Medical and Bio Allied Health Sciences Research, Ajman University, Ajman P.O. Box 346, United Arab Emirates; 3 College of Science, UAE University, Al Ain P.O. Box 15551, United Arab Emirates; alaajabberr@gmail.com

**Keywords:** canalicular adenoma, minor salivary gland neoplasms, immunohistochemistry

## Abstract

**Background/Objectives**: Canalicular adenoma (CaA) is a rare benign salivary gland neoplasm that primarily arises in minor salivary glands. Due to histological overlaps with other benign and malignant tumors, accurate diagnosis remains a challenge. This systematic review aims to comprehensively evaluate the clinical presentation, histopathological features, immunohistochemical (IHC) profiles, treatment modalities, and outcomes of CaA affecting the minor salivary glands. **Methods**: A systematic search of PubMed, Scopus, ScienceDirect, and Google Scholar was conducted for case reports and case series published in English between 2017 and 2025, following PRISMA guidelines. The review was registered in PROSPERO (CRD42023462417). Eligible studies described canalicular adenoma (CaA) of the minor salivary glands with clinical, histopathological, immunohistochemical, and treatment information. Risk of bias was assessed using the Joanna Briggs Institute tools, and data were synthesized descriptively due to heterogeneity in reporting. **Results**: Out of 1573 records identified, 23 studies met the inclusion criteria, comprising 44 cases. Canalicular adenoma most frequently affected the upper lip (63.6%), followed by the buccal mucosa and palate. Patients ranged from 29 to 84 years (mean 66 years), with a female predominance. Clinically, lesions presented as slow-growing, painless nodules measuring 2–40 mm. Histopathology consistently demonstrated branching canal-like structures lined by columnar or cuboidal epithelial cells, with immunohistochemistry showing positivity for AE1/AE3, CK7, S-100, and SOX10 and negativity for p63 and α-SMA. Surgical excision was the primary treatment, and recurrence was not reported in any of the 14 cases with documented post-treatment follow-up. Most studies showed moderate to high methodological quality. **Conclusions**: Canalicular adenoma is a rare benign neoplasm with a strong predilection for older women and for the upper lip. Accurate diagnosis requires correlation of clinical, histological, and immunohistochemical features due to its resemblance to other salivary gland tumors. Surgical management yields excellent outcomes, though multifocality may complicate interpretation of recurrence. Larger multi-institutional studies are needed to refine diagnostic criteria and clarify biological behavior.

## 1. Introduction

Canalicular adenoma (CaA) is a rare benign tumor of the salivary glands, affecting mainly the minor salivary glands, accounting for 0.5–12% of all minor salivary gland tumors and rarely presenting in the major salivary glands [1]. It was previously classified as a variant of basal cell adenoma, but this changed in 1991, when the WHO classified it as a distinct entity from other monomorphic adenomas, and that has been true until now [1].

CaA is usually present in patients within the fourth and seventh decades of life, rarely before the age of 50, and has a slight proclivity for women, with a female-to-male ratio of 1.7:1 [1]. Clinically, CaA presents in the minor salivary glands as an asymptomatic painless swelling, usually affecting the upper lip (80% of cases), followed by the buccal mucosa, with reports of it also affecting the palate, tongue, and rarely the parotid gland [1,2]. The lesion is usually small, ranging from a few millimeters to 2 cm in size. Due to its slow growth and lack of specific symptoms, the diagnosis of CaA is incidental during a routine dental examination. The clinical and histological presentation of CaA can resemble other salivary gland tumors such as basal cell adenoma, adenoid cystic carcinoma, or polymorphous adenocarcinoma [3,4]. The latter two are locally aggressive tumors that require extensive treatment, so appropriate differentiation between these lesions is critical.

Histological examination is imperative to be able to accurately diagnose CaA; the addition of immunohistochemical tests may assist in confirming the diagnosis in ambiguous cases. Microscopically, CaA typically presents as an encapsulated single or multifocal nodule/s, with a characteristic tubular or ductal architectural pattern. It is composed of one cell type, that is, columnar and/or cuboidal epithelial cells with monomorphic nuclei and eosinophilic cytoplasm [1,4]. These cells proliferate, forming single or double layers, arranged in anastomosing or branching patterns that sometimes join or separate in a paucicellular and vascular stroma. In some cases, cystic spaces/cavities may be observed that are filled with mucinous or eosinophilic material [1,5]. The treatment of choice for CaA arising from minor salivary glands is surgical excision, and the prognosis is generally favorable after complete removal. Recurrence rarely occurs with CaA, but is commonly seen in multifocal cases of CaA, and even then, it is difficult to verify if it is truly recurrence or due to its multifocality [5,6].

While previous reviews of canalicular adenoma exist, they encompass historical epochs with highly variable diagnostic criteria [5]. This systematic review provides a distinct update by focusing exclusively on minor salivary gland cases published between 2017 and 2025. Restricting our synthesis to this modern era aligns our dataset strictly with contemporary WHO classifications, capturing the precise diagnostic trends, multi-marker immunohistochemical profiles, and clinical behaviors that define modern oral and maxillofacial pathology practice [1]. The objective of this review is to evaluate the clinical picture of CaA in minor salivary glands to find trends in its presentation, to evaluate its histopathological characteristics to differentiate it from other benign or malignant lesions that have similar presentation, and to use immunohistochemistry to aid diagnosis, as well as to examine its treatment and outcome.

## 2. Materials and Methods

### 2.1. Protocol and Registration

This review was conducted following the Preferred Reporting Items for Systematic Reviews and Meta-Analyses (PRISMA) guidelines, CaA PRISMA checklist File S1 in supplementary, in order to ensure transparency and clarity throughout the reviewing process [7]. It was also prospectively registered in PROSPERO (ID: CRD42023462417).

### 2.2. Research Strategy

We conducted a comprehensive literature search of case reports published in PubMed, Scopus, ScienceDirect, and Google Scholar. The search was limited to reports published between 2017 and 2025, following the most up-to-date classification of salivary gland tumors of the World Health Organization (2017), and articles in the English language [1]. The literature search used targeted phrases for PubMed, Scopus, ScienceDirect, and Google Scholar, which are presented in Supplementary Table S1. The final updated search was performed in November 2025. The database filters used included date restriction from 2017 to 2025 and articles only in the English language.

Records were initially reviewed by title and abstract by two independent reviewers (N.A. and M.A.). While maintaining a list of excluded studies and updating to ensure no selection bias, the full texts of the selected studies were assessed for eligibility. When the reviewers had differing views, they resolved them through discussion; if consensus was not reached, a third reviewer (M.J.) was consulted.

### 2.3. Eligibility

Inclusion criteria

The articles included in this review meet the following criteria:

(i) Case reports and case series describing canalicular adenoma in human subjects.
(ii) Full-text availability
(iii) In English only, or with available English translation.
(iv) Between the years 2017 and 2025.
(v) Sufficient core case information presented to allow data synthesis: confirmed histopathological diagnosis of canalicular adenoma along with essential demographic and clinical features. Studies were not excluded solely due to isolated missing secondary metrics.

Exclusion criteria

Articles were excluded if they were:

(i) Publications such as review articles, editorials, animal studies, and conference abstracts.
(ii) Studies on other tumors in minor salivary glands.
(iii) Non-English articles.
(iv) Duplicates that may introduce bias.
(v) Articles published before 2017.
(vi) Articles with incomplete demographic, histopathological, and clinical details.

### 2.4. Quality Assessment

The quality of the final list of included studies was independently assessed by two reviewers (N.A. and M.A.) using the Joanna Briggs Institute (JBI) Critical Assessment tool for case reports [8]. The tool focuses on sufficient demographics, history, presentation, diagnosis, and proper intervention. Studies scoring 7–8 were considered to be “reporting completeness”, 5–6 “moderate reporting completeness”, and <4 “low reporting completeness”. Unclear or missing clinical criteria were categorized as “Not Reported” within the appraisal matrix rather than acting as cumulative quality penalties.

### 2.5. Data Extraction

Prior to synthesis, the extracted data were standardized to ensure consistency between included case reports and case series. Only English-language studies reporting cases of canalicular adenoma, following the (2017) updated classification of CaA as a distinct separate entity from monomorphic adenoma, were included. This classification remains unchanged in the 2022 WHO edition [1]. Extracted data comprised author(s) and year of publication, patient demographics (age and sex), tumor location (primary site), clinical presentation, evolution time, tumor size, treatment approach, and follow-up outcomes [Table 1].

## 3. Results

### 3.1. Literature Search

**Figure 1:**
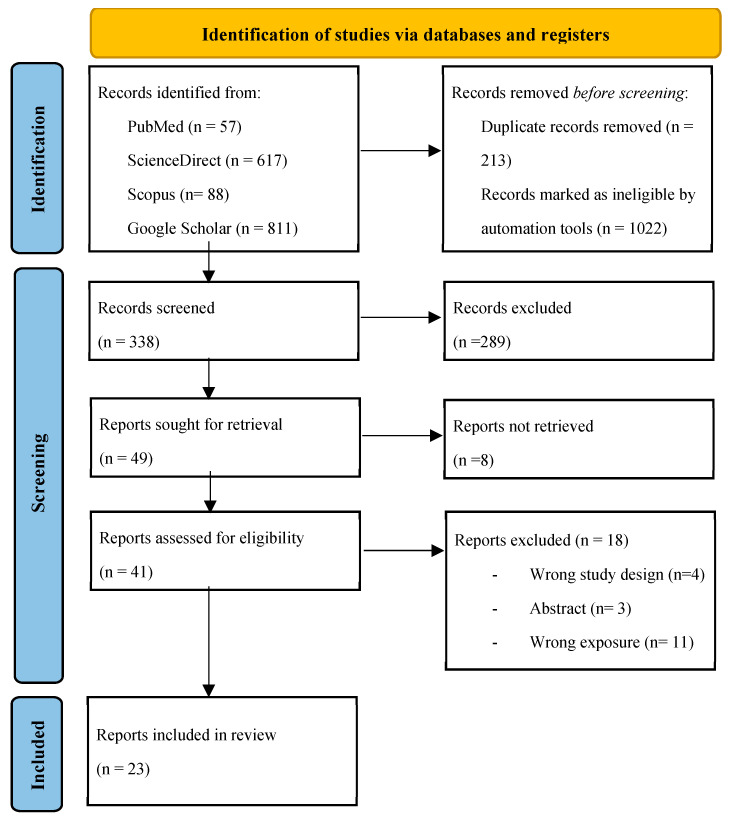
PRISMA flow diagram.

A total of 1573 records were identified through database searches. After removing duplicate records by exporting the search, importing it into Mendeley reference manager version 2.145.0, and filtering based on language and date restriction (2017–2025), 338 records remained for screening. Based on the title and abstract review, 289 records were excluded, leaving 49 full-text articles for eligibility assessment. Following a detailed review of the remaining 41 reports, 18 full-text articles were excluded; the reasons for exclusion are presented in Supplementary Table S2. Ultimately, 23 studies were included in the qualitative synthesis (Figure 1).

### 3.2. Study Characteristics

A total of 23 articles that included 44 patients met the inclusion criteria. The 23 articles were case reports and case series published between 2017 and 2025. Variables included sex, age, location of the tumor, duration of tumor evolution, clinical characteristics and size of the tumor, treatment provided and follow-up of recurrence [Table 1]. Additional information obtained included the histopathological details of the cases and the results of the immunohistochemical tests, if any were available.

### 3.3. Quality Assessment

It should be noted that JBI scores reflect reporting completeness rather than methodological strength or certainty of evidence, as presented in Table 2. Studies scoring 7–8 were considered to have “high reporting completeness”, those scoring 5–6 were considered to have “moderate reporting completeness”, and those with scores < 4 were considered to have “poor reporting completeness”. Of the 23 articles, 13 scored high reporting completeness and 10 moderate reporting completeness. Moderate scores were due to a lack of data on some criteria [Table 2].

### 3.4. Comparative Analysis of the Studies (Table 1)

Patient age ranged from 29 to 84 years (mean: 66 years). Most patients were older adults, with relatively few cases reported below 50 years of age. A female predominance was observed, accounting for 61.7% of cases (male-to-female ratio of 1:1.6).

Regarding the primary site of the tumor, the upper lip was the most commonly affected, accounting for 63.6% (27/44) of cases, followed by the buccal mucosa in 20.5% (9/44) and the palate in 13.6% (6/44). Less frequent sites included the tongue (2.3%, 1/44). Tumor size ranged from 2 to 40 mm but showed a trend towards larger lesions in certain anatomical areas, such as the palate. Reported symptom duration ranged from 1 month to 10 years. Clinically, most often presented as a well-circumscribed nodule (32 cases, 72.7%), usually described as firm or hard, while 12 cases were reported primarily as swellings (27.3%). The majority of lesions were painless or non-tender (47.7%) and asymptomatic (31.8%). Pain or sensitivity was uncommon, reported in only two cases, while mild discomfort was noted in three cases. Mobility was documented in nine cases, and overlying mucosa was typically normal; however, bluish or reddish discoloration was described in a minority of cases, particularly in larger or long-standing lesions. Treatment was most commonly excision of the lesion in more than 65% of the lesions, while in some cases, only incision was performed. Only 14 of the included articles followed patients after treatment, reporting no recurrence (NR) of the lesion (31.8%).

### 3.5. Histopathological Findings (Supplementary Table S3)

Histopathological examination demonstrated that canalicular adenomas were predominantly well-circumscribed lesions. Microscopically, tumors were composed of cuboidal to columnar or tall cylindrical epithelial cells with eosinophilic cytoplasm and round to oval, occasionally hyperchromatic nuclei, arranged in single or double cell layers. Mitotic figures were rare.

The characteristic architectural pattern consisted of canal-like ductal structures, formed by branching, anastomosing cords and tubules, frequently producing a beading or party-wall appearance. Cystic or pseudocystic spaces were commonly observed and occasionally contained hemorrhage, eosinophilic secretions, or squamous morules.

The stroma was most often loose, paucicellular, and highly vascular, with reported variations including fibrocollagenous, myxoid, or fibromyxoid backgrounds. Focal stromal changes such as edema, inflammatory infiltrates, and extravasated red blood cells were noted in selected cases. No features of malignancy, including perineural invasion or necrosis, were identified. Overall, despite morphologic variability, the histopathological findings were consistent with a diagnosis of canalicular adenoma.

### 3.6. Immunohistochemical Findings (Supplementary Table S4)

1. Positive immunohistochemical markers:
  - Neural Crest and Epithelial Markers: S100 was the most consistently positive marker, with 68.2% (30/44) of cases showing strong positivity and 2.3% (1/44) showing focal positivity. CK7 followed closely, with 61.4% (27/44) of cases demonstrating positivity.
  - Other Positive Markers: CD117 was positive in 31.8% (14/44) of cases. SOX10 and p16 also showed significant expression, with positivity rates of 25% (11/44) and 22.7% (10/44), respectively.
2. Negative immunohistochemical markers:
  - Myoepithelial Exclusion: Markers typically associated with myoepithelial cells were notably negative, including α-SMA (38.6% negative), calponin (29.5% negative), p40 (27.3% negative), and p63 (40.9% negative). Only a single case (2.3%) showed focal positivity for p63.
3. Varied Findings:
  - GFAP Heterogeneity: Unlike the strictly negative results often reported in the general literature, 22.7% (*n* = 10) of cases in this study exhibited focal positivity, while 31.8% (*n* = 14) remained completely negative.
  - Vimentin and β-Catenin: Vimentin expression was diverse, with 18.2% (*n* = 8) positive and 2.3% (*n* = 1) showing focal staining. Similarly, β-Catenin was positive in 15.9% (*n* = 7) and focal in 9.1% (*n* = 4) of cases.
  - Cytokeratin Variation: Beyond the consistent CK7, other cytokeratins showed varied expression; CK14 and CK8 demonstrated focal positivity in 13.6% (*n* = 6) and 6.8% (*n* = 3) of cases, respectively.
  - Myoepithelial Focalism: Although markers like p63 were predominantly negative (40.9%, *n* = 18), rare focal positivity was recorded in 2.3% (*n* = 1) of the cohort, highlighting minor variations in the typical CaA profile.

## 4. Discussion

CaA is a very rare entity, accounting for approximately 1–3% of all salivary gland tumors [1,31,32]. Among over 12,000 salivary neoplasms at the AFIP (Armed Forces Institute of Pathology), CaA represented only about 2% of cases. Its reported frequency in studies of minor salivary gland tumors ranges from 6% to 9.2% [33,34,35,36]. Calculating a true incidence is challenging due to a lack of comprehensive data.

A clear age-related predilection was observed, with most cases occurring in the 6th-8th decades of life and a mean patient age of 66 years. This indicates that canalicular adenoma of minor salivary glands predominantly presents in elderly patients, consistent with its classification as a tumor of later adulthood, a feature that may assist clinicians in narrowing the differential diagnosis of submucosal lesions in elderly patients [3,4,5,37]. In addition, a moderate female predominance (male-to-female ratio of 1:1.6) was identified, consistent with previous descriptions in the literature. While the biological basis for this gender inclination remains unclear, hormonal or genetic influences have been postulated but not substantiated, warranting cautious interpretation and further investigation [31,34,35,36].

Anatomically, the upper lip was the most frequently involved site, accounting for nearly 60% of cases, followed by the buccal mucosa and palate. The consistency of these findings across included studies supports earlier observations regarding the typical presentation of canalicular adenoma [5,35,36,37,38,39]. This strong site predilection is clinically important, as painless upper lip nodules in older patients are often initially presumed to be mucoceles, fibromas, or other reactive lesions [3,4]. This further emphasizes that CaA should be routinely considered in the differential diagnosis of persistent, well-circumscribed upper lip masses, particularly when growth is slow and the overlying mucosa remains intact. The wide range in symptom duration, extending up to 10 years in some cases, further reflects the indolent nature of the tumor and the risk of delayed diagnosis. Patients typically presented with a slow-growing, painless (47.7%), firm, and mobile nodule. Many patients are asymptomatic (31.8%), while others present with discomfort (28.7%) and some reports of pain (4.5%) [40,41,42,43].

The microscopic data extracted from our cases show that the tumor is often encapsulated and multinodular, with a bosselated or lobulated periphery [37,38,39,40]. The microscopic architecture is characterized by a canalicular pattern—interanastomosing cords and tubules of bland basaloid cells with distinctive “beading” or “party-wall” appearance where parallel rows join. Cystic degeneration with papillary infolding is very common [37,38,39,40,41]. Unique features often present include luminal squamous balls/morules, which are thought to represent metaplasia, and microliths (calcifications) similar to sialoliths, which form from degraded mucosubstances enriched with calcium [42]. The stroma is typically loose, myxoid, hypocellular, and highly vascularized. The lesional cells are columnar or cuboidal, exhibiting minimal pleomorphism, delicate stippled chromatin, and inconspicuous nucleoli. Mitoses are rare, and necrosis is absent. While our review shows a highly uniform presentation, the broader clinicopathological literature notes that secondary metaplastic variations—such as the formation of luminal squamous morules or calcium-enriched microliths—can occasionally occur due to the degeneration of luminal mucosubstances. Identifying these classic architectural traits is vital; structural mimics like basal cell adenoma lack the paucicellular vascular stroma and classic “beading” arrangement, while malignant entities like adenoid cystic carcinoma (ACC) or polymorphous adenocarcinoma (PAC) exhibit infiltrative patterns, cytological atypia, and perineural invasion that were completely absent in our modern case sample.

A strict case-level analysis of the immunohistochemical (IHC) data within our review reveals a luminal epithelial lineage without myoepithelial differentiation across the contemporary case reports. Within the cases that explicitly reported IHC testing, the tumor consistently shows positive expression for pancytokeratin (AE1/AE3), CK7, and S100. Additionally, nuclear positivity for SOX10 was a uniform finding among the case reports that utilized it, alongside case-level documentation of CD117 (c-kit) and Vimentin expression [38,39,40]. Conversely, myoepithelial markers—specifically smooth muscle actin (α-SMA) and calponin—along with nuclear p63 and p40, were consistently reported as negative across the extracted study datasets [41,42,43,44,45]. The low Ki-67 proliferative index, when explicitly reported in the cases, further corroborates the tumor’s indolent biological behavior.

These findings align with classic immunophenotypic baselines, which establish that CaA exhibits strong S100 and nuclear SOX10 positivity alongside reactivity for low-molecular-weight cytokeratins and E-cadherin. The literature underscores that a distinctive linear reaction at the tumor–stroma interface is a classic diagnostic hallmark [3,28,29,30]. Furthermore, while normal salivary acini and intercalated ducts demonstrate nuclear p63 expression, CaA tumor cells characteristically display cytoplasmic staining or complete nuclear negativity. Ultimately, the consistent absence of true myoepithelial markers (p63, p40, SMA, and calponin) remains the single most valuable benchmark for distinguishing CaA from its closest morphological mimics, including basal cell adenoma, pleomorphic adenoma, and polymorphous adenocarcinoma (PAC) [39,40,42].

Accurate diagnosis requires distinguishing CaA from other salivary gland tumors based on histology and IHC, including basal cell adenoma, which lacks the canalicular pattern and beading, shows a dual cell population and is positive for myoepithelial markers. Pleomorphic Adenoma (PA) contains a chondromyxoid matrix and myoepithelial cells. Adenoid cystic carcinoma (ACC) has a biphasic cell population, infiltrative growth, prominent perineural invasion, and is typically p63 positive. Polymorphous Low-Grade Adenocarcinoma (PLGA) shows infiltrative growth, perineural invasion, and more vesicular nuclei. Other considerations include ductal adenoma, reticulated myoepithelioma, and odontogenic tumors like ameloblastoma and adenomatoid odontogenic tumor [40,41,42,43,44].

Surgical excision was the primary treatment strategy deployed across the reviewed literature (68.2%, 30/44 cases). Superficial lesions are often managed with simple enucleation, while a surgical excision with a small cuff of normal tissue is recommended for deeper or larger tumors due to the potential for multifocality and a bosselated border [44,45]. However, one challenge in managing this tumor lies in its multifocal growth pattern. It is often difficult to determine whether a recurrence of canalicular adenoma is a true recurrence or a result of multinodularity [6,42,43,44,45,46]. Recurrences are probably more likely to represent multifocal tumors, with conservative surgery the treatment of choice.

While the general historical literature attributes an excellent prognosis to CaA, robust prognostic estimates cannot be generated directly from our systematic review dataset due to incomplete long-term follow-up tracking. Follow-up documentation was absent in 68.2% of the cases. Among the 14 cases with available post-operative data, no recurrences were reported within follow-up intervals that were often short (ranging from 1 month to 3 years). Thus, while surgical management remains the clinical standard, the lack of reported recurrences must be interpreted within the context of incomplete and abbreviated follow-up reporting. Long-term follow-up is recommended, and the vast majority of patients are alive with no evidence of disease.

The current study of CaA is constrained by its reliance on case reports and case series, which are generally regarded as the least robust forms of evidence in the realm of evidence-based research. While these reports offer valuable narrative insights into the trends pertaining to CaA, including its characteristics, incidence, location, and biological behavior, as well as details about the affected demographics, such as gender, ethnicity, and age, they suffer from limitations that impede comprehensive statistical analysis. Consequently, the capacity for meaningful statistical inference is hindered. Additionally, the restriction to English-language publications may have introduced language bias by excluding potentially relevant case reports published in other languages. This may affect the representativeness of estimates of anatomical distribution, patient demographics, and clinical variability, particularly in populations where non-English-language journals predominate. Many case reports lack comprehensive details, resulting in incomplete information that may overlook significant observational trends. Notably, pertinent information such as patient ethnicity, gender, age, lesion size, and location was found to be missing in several of the reviewed cases. Additionally, the incidence of CaA might be underestimated due to the use of diverse terminologies and evolving classification systems over time. In essence, while commonalities exist across these studies in terms of certain clinical features and treatment approaches for CaA, there are considerable variations in lesion characteristics, sizes, and anatomical distribution, indicating the complexity and heterogeneity of these conditions. These variations might be influenced by factors such as geography, patient demographics, and specific pathological processes underlying these lesions.

Despite all the above-mentioned limitations, this study provides a comprehensive overview of CaAs involving MSG, highlighting their diverse clinical features, locations, and management approaches across different studies conducted in various countries. The data gathered from these studies contributes to the understanding and management of such conditions in clinical practice.

Further detailed research on the pathogenesis and molecular biology of CaAs is required for progress in treatment and prognosis evaluation. A consensus on the standard approach for treatment has not yet been established due to the rarity of CaAs. Furthermore, future research should prioritize systematic, multi-institutional collaborations and the meta-analysis of larger patient cohorts to overcome these gaps and generate more robust, generalizable evidence.

## 5. Conclusions

Canalicular adenoma is a rare, benign salivary gland tumor with a predilection for the upper lip and minor salivary glands. Its diagnosis relies on a combination of clinical, histopathological, and immunohistochemical findings. Surgical excision remains the cornerstone of treatment, with an excellent prognosis and low recurrence rates. Continued research is essential to refine diagnostic criteria, understand molecular mechanisms, and optimize management strategies for this uncommon neoplasm.

## Figures and Tables

**Table 1:** Characteristics of included studies.

Author/Year	Country	Age (yrs)	Gender	Location	Duration	Clinical Features	Size (mm)	Treatment	Outcome
Ordioni et al. 2017 [9]	France	61	M	Upper lip	2 years	Hard mobile mucosal nodule Normal overlying mucosa	15	Excised	3 years No recurrence
Pereira da Silva et al. 2017 [10]	Brazil	75 and 56	1 F 1 M	**Case 1 (F):** Upper lip **Case 2 (M):** Buccal mucosa	**Case 1:** 6 months **Case 2:** 1 years	Asymptomatic firm/fibrous submucosal nodule with smooth surface	20	Excised	N/R
Ortega et al. 2018 [11]	Brazil	61	F	Upper lip	12 months	Asymptomatic bilateral nodules, firm to fluctuant Normal overlying mucosa	2–8	Incised	2 years No recurrence
Phore and Singh 2018 [12]	India	38	F	Upper lip	1 year	Asymptomatic soft swelling, with bluish tinge + smooth and rubbery nodules Facial asymmetry	N/R	Excised	2 months No recurrence
Panhotra et al. 2020 [13]	India	65	F	Upper lip	8 months	Painless, firm swelling, with smooth surface	20	Excised	N/R
Belmehdi and El Harti 2020 [14]	Morocco	70	M	Buccal mucosa	5 months	Asymptomatic, well-defined, smooth and firm nodule Discomfort	10	Excised	1 year No recurrence
Harada et al. 2020 [15]	Japan	79	F	Buccal mucosa	3–4 years	Painless, mobile, soft and elastic mass on the buccal mucosa Asymptomatic submucosal nodules on the upper lip	Buccal case: 20 × 18 × 15 Upper lip case: 5 and 7	Excised	5 months No recurrence
Vidyadhari et al. 2020 [16]	India	55	F	Palate	3 months	Solitary well-defined swelling, soft to firm in consistency Normal overlying mucosa	40 × 40	Incised	N/R
Pettas et al. 2021 [17]	Greece	74 and 78	2 F	Case 1: Upper lip Case 2: Buccal mucosa	Several months	**First case**: Painless, slow-growing, bluish fluctuant submucosal mass **Second case**: Asymptomatic, bilateral, movable soft tissue masses, normal mucosa	7–10	Excised	N/R
Yadav et al. 2021 [18]	India	41	M	Tongue	1 month	Well-circumscribed firm swelling with smooth surface, non-tender, freely movable Normal overlying mucosa Mild discomfort	10 × 8 × 7	Excised	6 months No recurrence
Czarny et al. 2021 [19]	France	80	F	Upper lip	4 months	Well-circumscribed firm nodule	N/R	Excised	N/R
Panagiotis et al. 2021 [20]	Greece	84	M	Upper lip	N/R	Well-circumscribed, hard, painless, mobile submucosal swelling Normal overlying mucosa	7	Excised	No recurrence
Khodaei et al. 2021 [21]	Iran	29	F	Palate	N/R	Solitary nodule Normal overlying mucosa	3 × 5	Excised	6 months No recurrence
Sultan et al. 2021 [22]	Australia	80	F	Upper lip	N/R	Submucosal nodules, firm, waxy and mobile Normal overlying mucosa	7–11	Excised	1 month No recurrence
Nair et al. 2021 [23]	India	71	M	Palate	2 years	Well-defined painless nodule, slow-growing, non-tender, soft to firm in consistency Normal overlying mucosa	30 × 20	Excised	1 month No recurrence
Swamy et al. 2021 [24]	India	65	F	Buccal mucosa	2 years	Firm, movable growth, with bluish hue	25 × 20	Incised	N/R
Komatsu et al. 2022 [25]	Japan	81	F	Buccal mucosa	N/R	Soft elastic bulge with bleeding	30	Excised	3 months No recurrence
Kasthurirengan and John 2023 [26]	India	61	M	Palate	8 years	Painless, slow-growing, ulcerated swelling	21 × 9 × 6	Excised	6 months No recurrence
Al Wakeel and Musa 2023 [27]	Saudi Arabia	75	M	Buccal mucosa	Several years	Well-demarcated, hard swelling Discomfort	15 × 12 × 9	Excised	N/R
Carvalho et al. 2024 [28]	Brazil	Avg: 66.3 Range: 49–73	6 F 5 M	Upper lip: 10 Palate: 1	N/R	Painless nodules, two presenting with bluish hue, two presenting reddish, and the rest covered with normal mucosa	9–30	N/R	N/R
Papanikos et al. 2025 [29]	UK	68	F	Upper lip	10 yrs	Hard, painless, and mobile nodule Normal overlying mucosa	10	Excised	No recurrence
Aquino et al. 2025 [30]	Brazil	Avg: 61.2 Range: 49–71	6 F 4 M	Upper lip: 7 Buccal mucosa: 2 Palate: 1	1 mo–10 yrs	Eight asymptomatic cases Two pain/sensitivity Eight firm nodules Two soft nodules	3–25	Excised	N/R
Boyes et al. 2025 [31]	UK	76	F	Upper lip	1 year	Painless lumps	4–8 mm	Excised	1 year No recurrence
Total	10 countries	Avg: 66 Range: 29–84	F: 27 M: 17 Ratio: 1:1.6	Upper lip: 28 Buccal mucosa: 9 Palate: 6 Tongue: 1	1 mo–10 yrs	Nodule: 32 (usually hard/firm) Swelling: 12 Painless/non-tender: 21 Pain: 2 Asymptomatic: 14 Discomfort: 3 Mobile: 9	2–40	Excised: 30 Incised: 3	No recurrence: 14

N/R—Not Reported.

**Table 2:** Quality assessment of included studies.

Author/Year	Demographic	History	Clinical Condition	Diagnostic Tests	Treatment Described	Post-Treatment Condition	Recurrence	Summary	Overall Appraisal:
Ordioni et al. 2017 [9]	X	X	X	X	X	N/R	X	X	7
Pereira da Silva et al. 2017 [10]	X	X	X	X	X	N/R	N/R	X	6
Ortega et al. 2018 [11]	X	X	X	X	X	N/R	X	X	7
Phore and Singh 2018 [12]	X	X	X	X	X	N/R	X	X	7
Panhotra et al. 2020 [13]	X	X	X	X	X	N/R	N/R	X	6
Belmehdi and El Harti 2020 [14]	X	X	X	X	X	N/R	X	X	7
Harada et al. 2020 [15]	X	X	X	X	X	N/R	X	X	7
Vidyadhari et al. 2020 [16]	X	X	X	X	X	N/R	N/R	X	6
Pettas et al. 2021 [17]	X	X	X	X	X	N/R	N/R	X	6
Yadav et al. 2021 [18]	X	X	X	X	X	N/R	X	X	7
Czarny et al. 2021 [19]	X	X	X	X	X	N/R	N/R	X	6
Panagiotis et al. 2021 [20]	X	X	X	X	X	N/R	N/R	X	6
Khodaei et al. 2021 [21]	X	X	X	X	X	N/R	X	X	7
Sultan et al. 2021 [22]	X	N/R	X	X	X	N/R	X	X	6
Nair et al. 2021 [23]	X	X	X	X	X	N/R	X	X	7
Swamy et al. 2021 [24]	X	X	X	X	X	N/R	N/R	X	6
Komatsu et al. 2022 [25]	X	X	X	X	X	N/R	X	X	7
Kasthurirengan and John 2023 [26]	X	X	X	X	X	N/R	X	X	7
Al Wakeel and Musa 2023 [27]	X	X	X	X	X	N/R	X	X	7
Carvalho et al. 2024 [28]	X	X	X	X	N/R	N/R	N/R	X	5
Papanikos et al. 2025 [29]	X	X	X	X	X	X	X	X	8
Aquino et al. 2025 [30]	X	X	X	X	X	N/R	N/R	X	6
Boyes et al. 2025 [31]	X	X	X	X	X	X	X	X	8

N/R—Not Reported.

## Data Availability

No new data were created or analyzed in this study.

## Supplementary Materials

The following supporting information can be downloaded at https://www.mdpi.com/article/10.3390/jcm15124655/s1: Table S1. Search terms and filters used for each database; Table S2. Full-text exclusion reasons; Table S3. Histopathology reports for included studies; Table S4. Immunohistochemistry findings of included cases; File S1. CaA PRSIMA checklist. Refs. [2,47,48,49,50,51,52,53,54,55,56,57,58,59,60,61,62,63] are cited in the supplementary materials.

## References

[1] El-Naggar A.K., Chan J.K.C., Grandis J.R., Takata T., Slootweg P. (2017). WHO Classification of Head and Neck Tumours.

[2] Su V., Chen H., Khorsandi A., Chai R.L. (2023). A rare case of canalicular adenoma in the parotid gland: Highlighting diagnostic limitations of fine-needle aspiration. Am. J. Otolaryngol..

[3] Thompson L.D., Bauer J.L., Chiosea S., McHugh J.B., Seethala R.R., Miettinen M., Müller S. (2015). Canalicular adenoma: A clinicopathologic and immunohistochemical analysis of 67 cases with a review of the literature. Head Neck Pathol..

[4] Hellquist H., Paiva-Correia A., Vander Poorten V., Quer M., Hernandez-Prera J.C., Andreasen S., Zbären P., Skalova A., Rinaldo A., Ferlito A. (2019). Analysis of the clinical relevance of histological classification of benign epithelial salivary gland tumours. Adv. Ther..

[5] Peraza A.J., Wright J., Gómez R. (2017). Canalicular adenoma: A systematic review. J. Cranio-Maxillofac. Surg..

[6] Samar M.E., Avila E.R., Fonseca I.B., Anderson W., Fonseca G.M., Cantín M. (2014). Multifocal canalicular adenoma of the minor labial salivary glands. Int. J. Clin. Exp. Pathol..

[7] Page M.J., McKenzie J.E., Bossuyt P.M., Boutron I., Hoffmann T.C., Mulrow C.D., Shamseer L., Tetzlaff J.M., Akl E.A., Brennan S.E. (2021). The PRISMA 2020 statement: An updated guideline for reporting systematic reviews. BMJ.

[8] Moola S., Munn Z., Tufanaru C., Aromataris E., Sears K., Sfetcu R., Currie M., Qureshi R., Mattis P., Lisy K., Aromataris E., Munn Z. (2020). Chapter 7: Systematic reviews of etiology and risk. JBI Manual for Evidence Synthesis.

[9] Ordioni U., Campana F., Catherine J.H., Lan R. (2017). Canalicular adenoma of the upper lip: A short case study. MÉdecine Buccale Chir. Buccale.

[10] Silva L.P., Silva L.A.B., Silveira É.J.D., Oliveira P.T., Miguel M.C.C. (2017). Clinical, morphological and immunohistochemical findings in canalicular adenoma. J. Oral Diagn..

[11] Ortega R.M., Bufalino A., Almeida L.Y., Navarro C.M., Travassos D.C., Ferrisse T.M., Carlos R., León J.E. (2018). Synchronous polymorphous adenocarcinoma and canalicular adenoma on the upper lip: An unusual presentation and immunohistochemical analysis. Head Neck Pathol..

[12] Phore S., Singh R. (2018). Canalicular adenoma: A rare case report. CHRISMED J. Health Res..

[13] Panhotra S., Hassan M.J., Sehgal S., Khan S., Jetley S. (2020). Canalicular adenoma: A case report of a rare benign tumor of minor salivary gland of upper lip. Indian J. Pathol. Oncol..

[14] Belmehdi A., Harti K.E. (2020). Canalicular adenoma of oral mucosa. Int. J. Dent. Sci..

[15] Harada K., Tanaka S., Oya K., Uchihashi T., Yokota Y., Seki S., Fujishita Y., Kogo M. (2020). Multiple canalicular adenomas arising in the buccal mucosa and upper lip: Case report and literature review. J. Oral Maxillofac. Surg. Med. Pathol..

[16] Vidyadhari P., Tanveer S., Palakaturthi N. (2020). Canalicular adenoma of a minor salivary gland on the palate: A case report with review of literature. Eur. J. Biomed. Pharm. Sci..

[17] Pettas E., Theofilou V.I., Georgaki M., Daskalopoulos A., Kalyvas D., Lazaris A.C., Younis R.H., Nikitakis N.G. (2021). Canalicular adenoma with unicystic morphology. A rare entity. J. Clin. Exp. Dent..

[18] Yadav N., Khorate M., Figueiredo N. (2021). Canalicular adenoma of the tongue: Report of a unique case. Pan Afr. Med. J..

[19] Czarny K., Le Pelletier F., Ejeil A.L. (2021). A single firm nodule of the upper lip: A case report. Austin J. Clin. Pathol..

[20] Panagiotis K., Apostolos M., Eleftherios A., Athanasios P. (2021). Canalicular adenoma of minor salivary gland: Report of a case and a brief review of the literature. Stomatologija.

[21] Khodaei M., Amani M., Mirinezhad S., Rafieyan S. (2021). Canalicular adenoma of the hard palate: A rare case report. Dent. Res. J..

[22] Sultan A.S., Chang F.S.C., Cooper T., Jessri M. (2021). Synchronous multifocal canalicular adenomas. Head Neck Pathol..

[23] Nair P.K., Varma B.R., Veeraraghavan R., Janardhanan M. (2021). Canalicular adenoma: Palatal presentation of an uncommon lesion. BMJ Case Rep..

[24] Swamy L.H.R., Kokila G., Nataraj S., Praveen K.S., Kumaraswamy S., Laxmidevi B.L. (2021). Canalicular adenoma: A case with review. J. Dent. Sci. Res..

[25] Komatsu Y., Kawai T., Chiba T., Takeda Y., Yamada H. (2022). A case of canalicular adenoma with anemia. J. Surg. Case Rep..

[26] Kasthurirengan S., John R.S. (2023). An extremely rare case of synchronous low-grade polymorphous adenocarcinoma with canalicular adenoma of the minor salivary gland of the palate. Cureus.

[27] Alwakeel A., Shiekh Musa A. (2023). Canalicular adenoma in buccal space area: A case report. Int. J. Med. Sci. Clin. Invent..

[28] Carvalho M.V., Legarrea J.M.A., Ribeiro A.C.P., Fonseca F.P., de Andrade B.A.B., de Almeida O.P., Vargas P.A. (2024). Clinicopathological and immunohistochemical study of canalicular adenoma. Eur. J. Dent. Oral Sci. Diagn..

[29] Papanikos V., Kouroukli O., Kakouris V., Dais P. (2025). Differential diagnosis of a canalicular adenoma: A case report and literature review. Cureus.

[30] Aquino S.N., Bezerra H.K.F., Louredo B.V.R., Amaral-Silva G.K.D., Gaetti-Jardim E.C., Antunes D.M., Santos-Silva A.R., Lopes M.A., Vargas P.A. (2025). Clinicopathological and immunohistochemical aspects of conventional and unicystic canalicular adenoma: A case series. Oral Surg. Oral Med. Oral Pathol. Oral Radiol..

[31] Boyes H., Kumar R., Alemkunnapuzha M., Anabtawi M. (2025). A Case of Synchronous Bilateral Canalicular Adenoma and Polymorphous Adenocarcinoma of the Minor Salivary Glands of the Upper Lip. Cureus.

[32] Nelson J.F., Jacoway J.R. (1973). Monomorphic adenoma (canalicular type). Report of 29 cases. Cancer.

[33] Batsakis J.G. (1991). Pathology consultation oral monomorphic adenomas. Ann. Otol. Rhinol. Laryngol..

[34] Furuse C., Tucci R., Machado de Sousa S.O., Rodarte Carvalho Y., Cavalcanti de Araújo V. (2003). Comparative immunoprofile of polymorphous low-grade adenocarcinoma and canalicular adenoma. Ann. Diagn. Pathol..

[35] Pires F.R., Pringle G.A., de Almeida O.P., Chen S.Y. (2007). Intra-oral minor salivary gland tumors: A clinicopathological study of 546 cases. Oral Oncol..

[36] Waldron C.A., El-Mofty S.K., Gnepp D.R. (1988). Tumors of the intraoral minor salivary glands: A demographic and histologic study of 426 cases. Oral Surg. Oral Med. Oral Pathol..

[37] Fonseca F.P., Carvalho M.D.V., de Almeida O.P., Rangel A.L.C.A., Takizawa M.C.H., Bueno A.G., Vargas P.A. (2012). Clinicopathologic analysis of 493 cases of salivary gland tumors in a Southern Brazilian population. Oral Surg. Oral Med. Oral Pathol. Oral Radiol..

[38] Żurek M., Fus Ł., Niemczyk K., Rzepakowska A. (2023). Salivary gland pathologies: Evolution in classification and association with unique genetic alterations. Eur. Arch. Oto-Rhino-Laryngol..

[39] Rooper L.M., Agaimy A., Assaad A., Bal M., Eugene H., Gagan J., Bishop J.A. (2023). Recurrent IDH2 mutations in salivary gland striated duct adenoma define an expanded histologic spectrum distinct from canalicular adenoma. Am. J. Surg. Pathol..

[40] Speight P.M., Barrett A.W. (2020). Salivary gland tumours: Diagnostic challenges and an update on the latest WHO classification. Diagn. Histopathol..

[41] Ohtomo R., Mori T., Shibata S., Tsuta K., Maeshima A.M., Akazawa C., Watabe Y., Honda K., Yamada T., Yoshimoto S. (2013). SOX10 is a novel marker of acinus and intercalated duct differentiation in salivary gland tumors: A clue to the histogenesis for tumor diagnosis. Mod. Pathol..

[42] Huebner T.A., Almubarak H., Drachenberg C.B., Papadimitriou J.C. (2014). Canalicular adenoma—search for the cell of origin: Ultrastructural and immunohistochemical analysis of 7 cases and review of the literature. Ultrastruct. Pathol..

[43] Buchner A., Merrell P.W., Carpenter W.M. (2007). Relative frequency of intra-oral minor salivary gland tumors: A study of 380 cases from northern California and comparison to reports from other parts of the world. J. Oral Pathol. Med..

[44] Jones A.V., Craig G.T., Speight P.M., Franklin C.D. (2008). The range and demographics of salivary gland tumours diagnosed in a UK population. Oral Oncol..

[45] Sivolella S., Valente M., De Biagi M., Mazzoleni S.S., Tellini E. (2014). Canalicular adenoma immunoprofile: A case report. Gerodontology.

[46] Tyralik D., Dzierwa-Gawron A., Rys J. (2013). Canalicular adenoma of the upper lip. Metachronous (multifocal) canalicular adenoma of the upper lip: A case report of an unusual finding. Pol. J. Pathol..

[47] Alramadhan S.A., Fitzpatrick S.G., Cohen D.M., Bhattacharyya I., Islam M.N. (2020). Retrospective Study of Buccal Mucosal Salivary Neoplasms. Head Neck Pathol..

[48] Toper M.H., Sarioglu S. (2021). Molecular Pathology of Salivary Gland Neoplasms: Diagnostic, Prognostic, and Predictive Perspective. Adv. Anat. Pathol..

[49] Bruzinga F.F.B., Fernandes F.C.F., Dias F.R., Lima M.G., deSouza P.E.A., deAguiar M.C.F., Grossmann S.D.M.C. (2023). Clinical and demographic features of minor salivary gland tumors: A collaborative study of 480 cases. Oral Dis..

[50] Barca I., Ferragina F., Staglianò S., Tarallo G., Sottile A.R., Ioppolo M.G., Frasca M., Cristofaro M.G. (2025). Minor salivary gland tumors: A retrospective review of cases in a single centre of south Italy. Am. J. Otolaryngol..

[51] Mendes I.C., Barros C.C.S., Souza A.J.S., Medeiros R.C.T., Turatti E., Cavalcante I.L., Cavalcante R.B. (2020). Canalicular adenoma in upper lip: A case report in young patient. Oral Surg. Oral Med. Oral Pathol. Oral Radiol..

[52] Flavio D.E.D.L., De Oliveira S.P., Cavalcanti L., Agostini M., De Andrade B.A.B., Romañach M.J., Abrahão A.C. (2022). Canalicular adenoma: Report of two cases with unusual findings. Oral Surg. Oral Med. Oral Pathol. Oral Radiol..

[53] Bastos D.C., Bufalino A., Navarro C.M., Da Silva A.R., León J.E., de Almeida L.Y., Ortega R.M. (2017). Synchronous polymorphous low-grade adenocarcinoma and canalicular adenoma on the upper lip: An unusual presentation. Oral Surg. Oral Med. Oral Pathol. Oral Radiol..

[54] Katabi N., Sukhadia P., DiNapoli S.E., Weinreb I., Hahn E., Ghossein R., Xu B. (2024). Expanding the histological spectrum of salivary gland neoplasms with HMGA2::WIF1 fusion emphasising their malignant potential: A report of eight cases. Histopathology.

[55] Thangaraja A., Ramaswamy V., N T.B., Uthaiah S.B. (2023). Canalicular adenoma of parotid gland: A case report and review of literature. IP Arch. Cytol. Histopathol. Res..

[56] Kim D.H., Paeng J.Y., Lee S.T., Choi S.Y. (2017). Canalicular Adenoma of the Parotid Gland: A Rare Case Report and Review of Literature. J. Clin. Case Rep..

[57] Agaimy A., Ihrler S., Baněčková M., Costés Martineau V., Mantsopoulos K., Hartmann A., Iro H., Stoehr R., Skálová A. (2022). HMGA2-WIF1 Rearrangements Characterize a Distinctive Subset of Salivary Pleomorphic Adenomas with Prominent Trabecular (Canalicular Adenoma-like) Morphology. Am. J. Surg. Pathol..

[58] Muthukumar R., Rajasekaran S., Prabakaran S., Navin R.B.N., Balaji D., Gowthame K., Kumar B.S., Adithya V. (2024). Canalicular Adenoma of Parotid-A Rare Case Report. Indian J. Otolaryngol. Head. Neck Surg..

[59] Azar A., Alkheder A., Salam R., Elnasser M.S., Alahmad V., Hajjar F. (2024). Canalicular Adenoma in the Parotid Gland: A Rare Case Study. Ear Nose Throat J..

[60] Ray M., Sathe P., Ghodke R., Suryavanshi M. (2018). Canalicular adenoma arising from the nasal septum in a child: First case report. Indian J. Pathol. Microbiol..

[61] Martins-Chaves R.R., Avelar M.C.M., Ferreira A.L.D., de Almeida A.P., Gomes G.V.S., Neves P.L.A., Fonseca F.P., Gomez R.S. (2024). Striated Duct Adenoma: A Case Report and a Scoping Review. Head Neck Pathol..

[62] Chandwani S., Shah A., Mittal N., Bal M. (2022). Striated duct adenoma of the parotid: A potential diagnostic pitfall. Indian J. Pathol. Microbiol..

[63] Karmouch M.A., Benghaleb H., Bijou W., Oukessou Y., Rouadi S., Abada R., Roubal M., Mahtar M. (2024). Canalicular Adenoma in the Parapharyngeal Space: A Rare Case Report. Asian J. Case Rep. Surg..

