# Supplementary material for: Canalicular Adenomas of Minor Salivary Glands: A Systematic Review of Case Reports and Case Series
Source: J Clin Med. 2026 Jun 16;15(12):4655. doi: 10.3390/jcm15124655 (PMC13301677; doi:10.3390/jcm15124655)
Supplement: Supplementary file 1 [file jcm-15-04655-s001.zip › jcm-4351347-supplementary-corrected.pdf]

Supplementary Table S1. Search terms and filters used for each database.

Database	Search Strategy / Boolean String	Applied Filters / Limits	Records Retrieved
PubMed	"canalicular"[All Fields] AND ("adenoma"[MeSH Terms] OR "adenoma"[All Fields] OR "adenomas"[All Fields]) AND ("salivary glands, minor"[MeSH Terms] OR ("salivary"[All Fields] AND "glands"[All Fields] AND "minor"[All Fields]) OR "minor salivary glands"[All Fields] OR ("minor"[All Fields] AND "salivary"[All Fields] AND "glands"[All Fields]))	• Publication Date: 2017-2025<br>• Language: English	57
Scopus	All fields ("canalicular adenoma" AND "minor salivary gland" OR "oral")	• Year: 2017–2025<br>• Language: English	*88*
ScienceDirect	Title, abstract, keywords: "canalicular adenoma" AND "minor salivary"	• Year: 2017–2025<br>• Language: English	617
Google Scholar	"canalicular adenoma" "minor salivary glands" OR “intraoral”	• Custom Range: 2017–2025	811
Total Records			**1,573**

Supplementary Table S2. Full-text exclusion reasons.

Articles	Reason for exclusion
Alramadhan et al. 2020[47]<br>Toper & Sarioglu 2021[48]<br>Bruzinga et al. 2021[49]<br>Barca et al. 2025[50]	Wrong study design
Mendes et al. 2020[51]<br>Estanho et al. 2022[51]<br>Bastos et al. 2017[53]	Publication type: Abstract
Katbi et al. 2024[54]<br>Su et al. 2023[2]<br>Thangaraja et al. 2023[55]<br>Kim et al. 2017[56]<br>Agaimy et al. 2022[57]<br>Muthukumar et al. 2024[58]<br>Azar et al. 2024[59]<br>Ray et al. 2018[60]<br>Martins-Chaves et al. 2024[61]<br>Chandwani et al. 2022[62]<br>Karmouch et al. 2024[63]	Wrong exposure

Supplementary Table S3. Histopathology reports for included studies.

Author/Year	Macroscopically	Microscopically		
	Well-circumscribed	Columnar &/or cuboidal cells	Stroma	Arrangement
**Ordioni et al. 2017** [9]	Yes	Yes	Loose, paucicellular, & rich with blood capillaries	Canaliculi appearance<br>Number of mitoses low
**Pereira da Silva et al. 2017** [10]	Yes	Yes	Paucicellular & highly vascularised	Beaded anastomosing bilayered strands & cords
**Ortega et al. 2018** [11]	-	Yes	Loose vascular	Canali-like structures<br>Beading appearance<br>Intraluminal squamous balls or morules
**Phore & Singh 2018** [12]	-	Yes	Loose, paucicellular & vascular	Branching and anastomosing canalicular structure
**Panhotra et al. 2020** [13]	Yes	Yes	Fibrocollagenous, vascular, & inflammatory infiltrate	Branching and anastomosing canalicular structure
**Belmehdi & El Harti 2020** [14]	Yes	Yes	-	Lobulated with nodule
**Harada et al. 2020** [15]	Yes	Yes	Loose & vascular	Cord-like or tubular structures<br>Beadlike appearance with cell aggregates<br>Cystic cavities
**Vidyadhari et al. 2020** [16]	-	Yes	Scanty	Long cords in parallel forming canals<br>Party wall appearance<br>Few cystic spaces
**Pettas et al. 2021** [17]	Yes	Yes	Case 1: Paucicellular & fibrous<br>Case 2: Inconspicuous	Solid or trabecular formation<br>Focal areas of solid arrangement<br>Cystic spaces
**Yadav et al. 2021** [18]	Yes	Yes	-	Canal like ductal structure
**Czarny et al. 2021** [19]	Yes	Yes	Loose with few cells	Anastomosing tubes<br>Beading pattern
**Panagiotis et al. 2021** [20]	Yes	Yes	Myxoid	-
**Khodaei et al. 2021** [21]	-	Yes	Hypocellular & vascular	Lobular islands<br>Parallel rows of cells forming ductal structures
**Sultan et al. 2021** [22]	Yes	Yes	Loose hypocellular myxoid	Tubular growth<br>Canalicular morphology seen<br>Cystic changes with hemorrhage seen<br>Intraluminnal squamous morules seen
**Nair et al. 2021** [23]	Yes	Yes	Scanty & vascular	Bilayered appearance

				Luminal spaces with eosinophilic secretions with areas of hyalinisation
**Swamy et al. 2021 [24]**	Yes	Yes	Fibrocollagenous, extravasated RBC's & adipose tissue in focal area	Canalicular arrangement<br>Party wall appearance & duct-like pattern
**Komatsu et al. 2022 [25]**	Yes	Yes	-	Branching & interconnecting canalicular strands<br>Cystic structures
**Kasthurirengan & John 2023 [26]**	Yes	Yes	Loose, oedematous, vascular, & inflammatory infiltrate	Branching & anastomosing cords and tubules
**Al Wakeel & Musa 2023 [27]**	Yes	Yes	Loose, fibrous, & vascular	Bilayered appearance<br>Tubular growth pattern<br>Cyst formation<br>Intraluminnal Squamous ball or morule
**Carvalho et al. 2024 [28]**	Yes	Yes	Loose, fibrillar material with vascular background	Canaliculi appearance<br>Beading appearance<br>Pseudocystic spaces<br>Foamy macrophages seen
**Papanikos et al. 2025 [29]**	Yes	Yes	Oedematous, with myxoid and sclerosing areas<br>Intraluminal haemorrhage inside the cystic spaces	Anastomosing cords and strands with tubule & cyst formation<br>Beading appearance
**Aquino et al. 2025 [30]**	Yes	Yes	Fibrillar, sparse, and edematous,	Canaliculi, strands, budding or branching cords or tubules<br>Solid areas seen<br>Beading & lobulated appearance<br>Cystic formations + intraluminal haemorrhage

Supplementary Table S4. Immunohistochemistry findings of included cases.

Marker	Positive		Focal positive		Negative	
	n	%	n	%	n	%
S100	30	68.2	1	2.3	0	0
CK7	27	61.4	0	0	0	0
SOX10	11	25	0	0	0	0
p40	0	0	0	0	12	27.3
p63	0	0	1	2.3	18	40.9
p16	10	22.7	1	2.3	0	0
GFAP	0	0	10	22.7	14	31.8
CD117	14	31.8	0	0	0	0
SMA	0	0	0	0	17	38.6
Vimentin	8	18.2	1	2.3	5	
Pan-cytokeratin	4	9.1	0	0	0	0
Calponin	0	0	0	0	13	29.5
CK 13	6	13.6	3	6.8	2	4.5
CK 14	5	11.4	6	13.6	1	2.3
CK 8	7	15.9	3	6.8	2	4.5
B-Catenin	7	15.9	4	9.1	1	2.3
